# The Anti-Adhesive Effect of Curcumin on *Candida albicans* Biofilms on Denture Materials

**DOI:** 10.3389/fmicb.2017.00659

**Published:** 2017-04-20

**Authors:** Hasanain Alalwan, Ranjith Rajendran, David F. Lappin, Emilie Combet, Muhammad Shahzad, Douglas Robertson, Christopher J. Nile, Craig Williams, Gordon Ramage

**Affiliations:** ^1^Oral Sciences Research Group, Glasgow Dental School, University of GlasgowGlasgow, UK; ^2^Department of Prosthodontics, College of Dentistry, University of BaghdadBaghdad, Iraq; ^3^Department of Human Nutrition, School of Medicine, Dentistry and Nursing, College of Medical, Veterinary and Life Sciences, University of GlasgowGlasgow, UK; ^4^Institute of Basic Medical Sciences, Khyber Medical UniversityPeshawar, Pakistan; ^5^Institute of Healthcare Policy and Practice, University of West of ScotlandPaisley, UK

**Keywords:** Curcumin, polyphenol, *Candida albicans*, adhesion, adsorption

## Abstract

The use of natural compounds as an alternative source of antimicrobials has become a necessity given the growing concern over global antimicrobial resistance. Polyphenols, found in various edible plants, offers one potential solution to this. We aimed to investigate the possibility of using curcumin within the context of oral health as a way of inhibiting and preventing the harmful development of *Candida albicans* biofilms. We undertook a series of adsorption experiments with varying concentrations of curcumin, showing that 50 μg/ml could prevent adhesion. This effect could be further synergized by the curcumin pre-treatment of yeast cells to obtain significantly greater inhibition (>90%, *p* < 0.001). Investigation of the biological impact of curcumin showed that it preferentially affected immature morphological forms (yeast and germlings), and actively promoted aggregation of the cells. Transcriptional analyses showed that key adhesins were down-regulated (ALS1 and ALS3), whereas aggregation related genes (ALS5 and AAF1) were up-regulated. Collectively, these data demonstrated that curcumin elicits anti-adhesive effects and that induces transcription of genes integrally involved in the processes related to biofilm formation. Curcumin and associated polyphenols therefore have the capacity to be developed for use in oral healthcare to augment existing preventative strategies for candidal biofilms on the denture surface.

## Introduction

Increasingly there are fewer antimicrobial options available to treat life-threatening infections, due largely to therapeutic mismanagement actively driving resistance, coupled with disinvestment in antimicrobial drug development from the pharmaceutical industry. The antimicrobial resistance debate suggests an imminent return to an era of uncertainty and limited therapeutic options, suggesting that we should stop our wavering and tackle this threat head on.

Naturally derived chemotherapeutic agents are an attractive option, particularly those botanically derived molecules, which offer advantages over synthetic derivatives due to their natural evolution and diminished likelihood of resistance. An interesting active plant extract worth consideration is the polyphenol curcumin (diferuloylmethane), extracted from the rhizomes of the *Curcuma longa* plant (Mahmood et al., 2015). It is the active ingredient of turmeric and copiously used in Asia as a food additive or dietary supplement, though it forms typically < 5% composition (Esatbeyoglu et al., 2012; Kwon, 2014). Curcumin (CUR) shows safe and effective biological activities such as anti-inflammatory, antioxidant, anti-proliferation, with potential efficacy against many human diseases as suggested by animal studies (Gupta et al., 2012). Importantly, CUR displays broad-spectrum antimicrobial properties (Moghadamtousi et al., 2014), including antibacterial (Shahzad et al., 2015; Tyagi et al., 2015) and antifungal properties (Martins et al., 2009; Khan et al., 2012), as well as the ability to influence adhesive and biofilm properties (Shahzad et al., 2014, 2015).

Studies by our group have shown that CUR has the capacity to alter the adhesion of key periodontal pathogens, and impact overall biofilm formation (Shahzad et al., 2015). Parallel studies on the primary denture pathogen *Candida albicans* demonstrated that CUR exhibited anti-biofilm properties at high concentrations, as well as antifungal activity against planktonic and biofilm cells (Shahzad et al., 2014). It is thought that these elevated concentrations directly impact cell wall permeability through signaling of the MAP kinase and calcineurin-mediated signaling, pathways which maintains cell wall integrity (Kumar et al., 2014). *C. albicans* is a major global opportunistic pathogen, armed with recognized virulence determinants that include colonization factors (adhesins, hyphae and thigmotropic properties), as well as the release of invasins, such as hydrolytic proteins that facilitate invasion into the host (O'Donnell et al., 2015c). The ability to adhere to both biological and inert substrates and form biofilms makes this organism of particular interest in the context of oral disease (O'Donnell et al., 2016). Biofilm etiology in this environment is a primary mechanism of persistence and survival in the oral cavity, providing physical protection from endogenous and exogenous antimicrobial factors (Ramage et al., 2014). Most significantly, *C. albicans* prominent role in inducing inflammation to cause denture induced stomatitis means we have a keen interest in developing ways to manipulate and interfere with biofilm development, as this is critical in preventing this disease. Therefore, this study aimed to investigate whether CUR could be used through direct interaction with materials and the yeast *C. albicans* to interfere with early adhesion events on a clinically relevant substrate.

## Materials and methods

### Culture conditions and standardization

The laboratory based *C. albicans* SC5314 was used in this study (O'Donnell et al., 2016). Yeast cells were cultivated as working stocks on fresh Sabouraud agar (Sigma-Aldrich, UK) for 48 h at 30°C and maintained at 4°C. One unique colony was used to grow the cells in yeast-peptone-dextrose (YPD) medium (Sigma-Aldrich) for 18 h at 30°C and 150 rpm orbital shaker. The cells were washed twice by centrifuging in sterile phosphate buffered saline (PBS; Sigma-Aldrich, UK) and standardized using a Neubauer haemocytometer.

### Antifungal susceptibility testing

Planktonic and sessile cells were first investigated for their susceptibility to the polyphenol CUR (HPLC grade, Acros Organics, Belgium). Stock CUR was prepared immediately preceding the experiment using an non-antimicrobial concentration of dimethyl sulfoxide (DMSO) as a solvent and adjusted to < 5% v/v in RPMI-1640 medium (Sigma-Aldrich, UK; Shahzad et al., 2015). Standardized CLSI M-27A broth microdilution methodology was initially undertaken for planktonic yeast cultures in 96 well round bottomed microtitre plates (CLSI-M27-A, 2008). Clear wells with no visible growth were considered as the minimum inhibitory concentration (MIC). For sessile susceptibility testing, pre-formed 24 h biofilms were challenged with CUR using standardized sessile antifungal testing (Ramage et al., 2001; Pierce et al., 2008). Reduction of tetrazolium to formazan through an XTT assay was used, and the optical densities quantified at 492 nm using a microtitre plate reader (FluoStar Omega, BMG Labtech, UK). Negative and positive controls were included, and the experimental wells data were compared to the positive control data to reveal the SMIC_80_, where the optical density is reduced more than 80% in comparison to the positive control optical density, reflecting significant bioactivity against the biofilm. These procedures were repeated in three independent occasions where three replicates have been considered.

### Investigating the effect of CUR adsorption on adhesion

The potential capability of CUR to be adsorbed to denture material was investigated. Heat cure poly methyl methacrylate (PMMA) denture base material (Chaperlin and Jacobs Ltd, Surrey, UK) was used to fabricate 12 mm diameter discs using the dental compression molding technique. These discs were immersed in ddH_2_O for 7 days to ensure excess toxic monomers were removed. CUR was diluted in RPMI-1640 medium to 200, 400, and 800 μg/ml. Discs were distributed in 24 wells plates (Costar, Corning Incorporated, USA) and 1 ml of the CUR suspension was added. The plates were incubated at room temperature for a series of time points (1, 5, 10, 30, 60, 120, 240, and 1,440 min). Next, discs were transferred to fresh wells and washed with 1 ml of distilled water and then 1 ml of DMSO was added to dissolve the adsorbed CUR. To quantify the released CUR, a standard curve (0.39–100 μg/ml serially double diluted) was developed and measured at 436 nm using the spectrophotometer. Based on these data, PMMA discs were immersed in 1 ml of 800 μg/ml of CUR for 10 min (equivalent to 50 μg/ml) then washed with PBS to remove the unabsorbed molecules. These treated discs were inoculated with 1 ml of 5 × 10^5^ CFU of *C. albicans* SC5314 cells and incubated for 30 min at 37°C. Following the initial adhesion, cells were washed in PBS and the adherent cells removed by sonication at 35 kHz for 10 min (Ultrasonic bath, Fisher scientific, UK), and enumerated using the Miles and Misra plate counting method (Miles et al., 1938). The final cell number was expressed per cm^2^ PMMA, which was compared to a CUR negative control. All experiments were performed with three independent sections on three independent occasions. Scanning electron microscopy (SEM) was also performed using the same experimental parameters, then processed and imaged, as described previously by our group. Briefly, biofilms were grown on Thermanox™ coverslips or hydrogel cellulose matrix and treated, as previously described. Biofilms were washed twice with PBS, before being fixed in 2% para-formaldehyde, 2% glutaraldehyde, 0.15M sodium cacodylate, and 0.15% w/v alcian blue, at pH 7.4, and prepared for SEM as previously described (Erlandsen et al., 2004). The specimens were sputter-coated with gold and viewed under a JEOL JSM-6400 scanning electron microscope.

### Investigating the biological effect of CUR on adhesion and biofilm formation

Discs were adsorbed with CUR as described above, then the discs (untreated and treated) were inoculated with either 1 ml of 5 × 10^5^ CFU + 3 min CUR (50 μg/ml) or PBS (negative control) treated *C. albicans* SC5314 cells, and incubated for 30 min at 37°C. Adhesion of *C. albicans* was then assessed and quantified as described in the previous section, with the levels of adhesion expressed as a proportion of the negative control (PMMA-/*C. albicans*-). In parallel we then assessed whether longer CUR exposure time negatively impacted adhesion by treating cells for 3, 30, and 90 min, and the levels of adhesion to PMMA quantified. Finally, we assessed whether or not CUR exposure to *C. albicans* cells at different growth phases played a role, with the hypothesis that there may be differences in how yeast (Y), germlings (G), or hyphae (H) responded to this molecule. Briefly, cells were grown overnight, standardized to 1 × 10^6^ CFU in RPMI and inoculated into a 96-well microtitre plate. Cells were then exposed to 50, 100, or 200 μg/ml CUR at either 0 h (Y), 2 h (G), or 4 h (H) post-inoculation and incubated for a further 24 h at 37°C. Thereafter, the cells were washed in PBS and the resultant biofilm quantified using an XTT metabolic reduction assay. All experiments were performed in duplicate on three independent occasions.

### Investigating the aggregative effect of CUR

In order to assess whether CUR has additional effects on the physicality of these yeasts, we assessed its impact on aggregation. *C. albicans* SC5314 cells (Y) were standardized (1 × 10^6^ cells) in PBS and exposed ± to subinhibitory concentrations of CUR (50 μg/ml) for 90 min at 37°C under constant agitation (200 rpm). Following incubation the cells were serially 10 diluted in PBS diluent and plated onto Sabouraud agar using the Miles and Misra methodology. The plates were then incubated overnight at 37°C and the colonies enumerated. In parallel, cells were examined under a light microscope to evaluate aggregation visually. All experiments were performed in triplicate on three independent occasions.

### Investigating the molecular effect of CUR on adhesion and biofilm formation

Preparation of Y and H cells was performed using an initial inoculum of 1 × 10^8^ cells and 5 × 10^5^ cells of *C. albicans* SC5314 in RPMI, respectively. For H cells these were incubated on PMMA sections within 24 wells plates for 4 h. Both Y and H cells were then treated ± CUR (50 μg/ml) in RPMI for 3, 30, and 90 min, after which they were prepared for RNA extraction. Cells were either centrifuged or sonicated in a 35 kHz for 10 min (Ultrasonic bath, Fisher scientific, UK) to harvest the cells. These were then washed by centrifugation prior to RNA extraction using a combined mechanical disruption (0.5 mm glass beads) and chemical TRIzol™ method (Invitrogen, Paisley, UK). After DNase treatment (Qiagen, Crawley, UK) and purification (RNeasy MinElute clean up kit, Qiagen, Crawley, UK), cDNA was synthesized using a High Capacity RNA to cDNA kit (Life Technologies, Paisley, UK), and quantitative PCR performed using a SYBR® GreenER™ assay (Life Technologies Ltd, Paisley, UK). The primers used for quantitative PCR were ALS1, ALS3, ALS5 (agglutanin-like sequence 1, 3, and 5), EAP1 (epithelial adhesion protein 1), and AAF1 (adhesion and aggregation factor 1). Each parameter was analyzed in duplicate using MxProP Quantitative PCR machine and MxProP 3000 software (Stratagene, Amsterdam, Netherlands). Gene expression was normalized to the housekeeping gene ACT1 according to 2^−ΔΔCT^ method (Livak and Schmittgen, 2001). Table 1 summarizes all the primer details used in this study. A heatmap was created for the differential expression of genes (log_2_) over the period of 3–90 min from the untreated control compared to CUR exposed cells. Maps and clusters were generated in R with the use of heatmap 0.2 function from the gplots package. All experimnets were performed in triplicate on three independent occasions.

**Table 1:** **Primers used for real time qPCR transcriptional analysis of *Candida albicans***.

**Gene**	**Sequence (5′–3′)**	**References**
ALS1	F—TTCTCATGAATCAGCATCCACAA	Nailis et al., 2009
	R—CAGAATTTTCACCCATACTTGGTTTC	
ALS3	F—CAACTTGGGTTATTGAAACAAAAACA	Nailis et al., 2009
	R—AGAAACAGAAACCCAAGAACAACCT	
ALS5	F—CTGCCGGTTATCGTCCATTTA	Green et al., 2005
	R—ATTGATACTGGTTATTATCTGAGGGAGAAA	
EAP1	F—ACCACCACCGGGTATACAAA	Sherry et al., 2014
	R—GCCATCACATTTGGTGACAG	
AAF1	F—CTGCCCTTGTTGGTACATCT	This study
	R—TGGGATAGTTGGTGGAGGAG	
ACT1	F—AAGAATTGATTTGGCTGGTAGAGA	Ricardo et al., 2009
	R—TGGCAGAAGATTGAGAAGAAGTTT	

### Statistical analysis

As we were unable to ascertain that the data conformed to a Gaussian distribution data analysis was performed on non-parametric data using either a Mann–Whitney test or a Kruskal–Wallis test with Dunn's multiple comparison post-test. All independant data points are presented, with error bars representing the median with interquartile range. Where proportional data is presented, analysis was performed on the original data sets. All statistics and figures were produced using GraphPad Prism v.5 (GraphPad Software Inc., La Jolla, CA).

## Results

### Curcumin adsorption reduces *Candida albicans* adhesion

First, we tested the potential of CUR to inhibit and kill planktonic and biofilm cells to establish biologically active working concentrations suitable for use in downstream analyses. The planktonic MIC (PMIC) was shown to be 100 μg/ml of CUR for SC5314 and two other clinical strains tested (data not shown), whereas the sessile (biofilm) MIC that caused an 80% reduced metabolic activity (SMIC_80_) was ≥ 200 μg/ml, demonstrating that the biofilm's activity and/or viabilty are significantly reduced.

**Figure 1:**
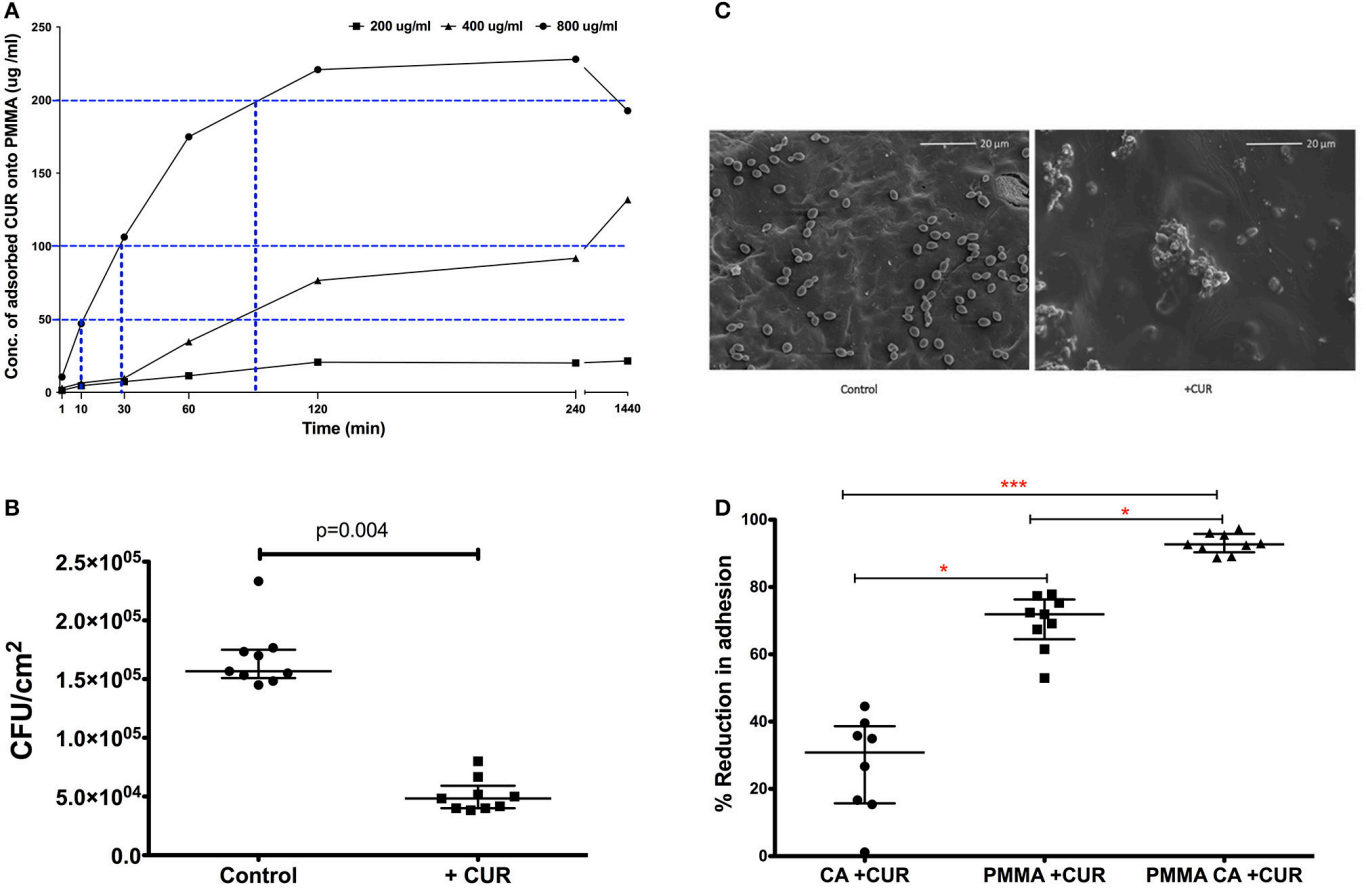
**The impact of CUR adsorption to PMMA and its impact on *Candida albicans* SC5314 adhesion. (A)** Time and concentration dependant adsorption of CUR to PMMA (blue dotted line) at half the MIC (50 μg/ml), MIC (100 μg/ml), and SMIC_80_ 200 μg/ml. **(B)** *C. albicans* inhibitory capability of 50 μg/ml adsorbed CUR onto PMMA compared to untreated control and a Mann–Whitney test was performed on data from nine independent experiments. **(C)** SEM images of 30 min adherent *C. albicans* cells onto CUR adsorbed (+CUR) and non-adsorbed PMMA control. **(D)** Single and dual-treatment of CUR on *C. albicans* adhesion, which were analyzed using a Kruskal–Wallis test with Dunn's multiple comparison post-test performed on data from nine independent experiments. All independant data points are presented, with error bars representing the median with interquartile range (^*^p < 0.05, ^**^p < 0.01, ^***^p < 0.001).

Based on these data we wanted to evaluate whether these levels of CUR could be adsorbed to PMMA material to prevent *C. albicans* adhesion. We therefore adsorbed 200, 400, and 800 μg/ml of CUR to PMMA sections over different time periods and quantified these adsorbed concentrations using an elution method alongside an optimized standard curve. Figure 1A illustrates the kinetics of adsorption for each concentration. It was shown that 800 μg/ml CUR was required to achieve concentrations with anti-biofilm activity (200 μg/ml), though 90 min adsorption was required to achieve this. Nevertheless, after 10 and 30 min adsorption, 50 and 100 μg/ml concentrations were achieved from this initial concentration, respectively. The lower concentration of 400 μg/ml was able to achieve PMIC levels, though this took ~90 min adsorption. Finally, 200 μg/ml was not able to achieve any antimicrobial level concentrations, even after 24 h adsorption. Based on these data we focussed on adsorption of 800 μg/ml for 10 min, which was able to achieve 50 μg/ml on the surface of the PMMA for downstream analysis. Next, we evaluated the capacity of *C. albicans* to attach to PMMA for 30 min adsorbed with CUR (50 μg/ml) and compared a control (Figure 1B). Here we showed a significant three-fold reduction in adhesion of *C. albicans* was observed (*p* < 0.004). When analyzed by SEM the visible reduction of yeasts cells can be shown on the PMMA surfaces (Figure 1C).

Given that CUR was shown to elicit antimicrobial effects, both planktonically and to a lesser extent against sessile biofilms cells, and that adsorption appeared to influence adhesive capacity, we decided to investigate whether combining an early direct effect on *C. albicans* with that of adsorption to PMMA would synergize CUR activity in prevention of adhesion and colonization. *C. albicans* was treated for 3 min ± CUR 50 μg/ml (CA + CUR), which was compared to both PMMA adsorbed with CUR (PMMA + CUR), or a combination of CUR treated *C. albicans* and CUR adsorbed PMMA (PMMA CA + CUR). Figure 1D demonstrates that the direct treatment of *C. albicans* alone only inhibited 30 min adhesion by 27%, which was significantly lower than CUR adsorption alone (70%, *p* < 0.05). However, combining the effect on *C. albicans* with adsorbed CUR resulted in a significant reduction than adsorption alone of 93% (*p* < 0.001), thus improving the anti-adhesive capacity of *C. albicans*.

### Curcumin prevents biofilm formation and promotes *Candida albicans* aggregation

**Figure 2:**
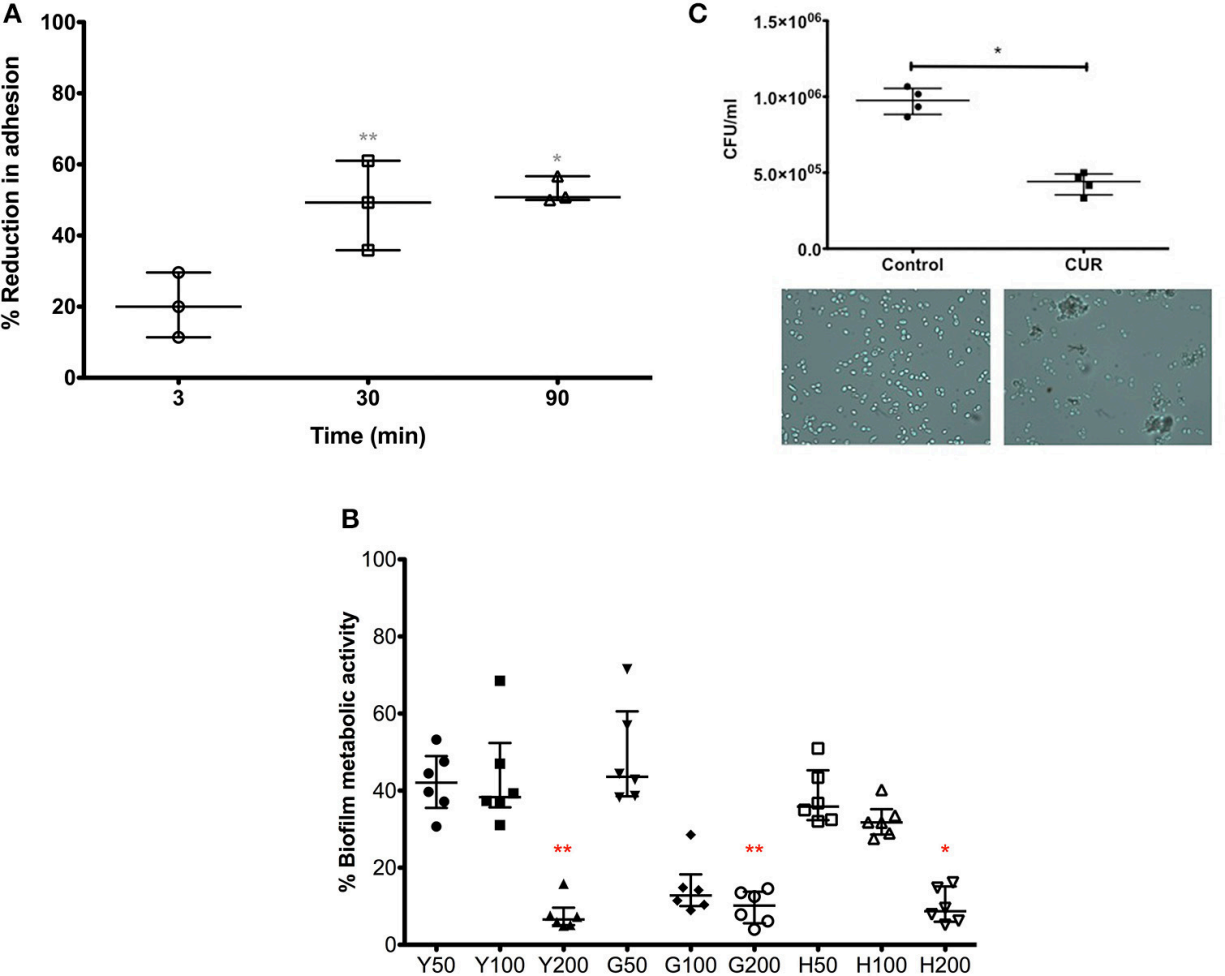
**The impact of CUR on *C. albicans* adhesion, biofilm formation, and aggregation. (A)** *C. albicans* was pre-treatment with sub-inhibitory concentration of CUR (50 μg/ml) for 3, 30, and 90 min and adhesion to PMMA assessed on data from triplicate data from three independent experiments. **(B)** Different morphological forms of *C. albicans* (Y, yeast; G, germlings; H, hyphae) were pre-treated with CUR at 50, 100, and 200 μg/ml, and the resultant biofilm formation assessed metabolically after 24 h. Data represents six independent experiments, which was analyzed using a Kruskal–Wallis test with Dunn's multiple comparison post-test. **(C)** Aggregation of *C. albicans* exposed to CUR (50 μg/ml) was assessed by total viable cell counts, analyzed using a Mann–Whitney test on triplicate data from four independent experiments, and the phenotype validated by light microscopy (400 × magnification). All independent data points are presented, with error bars representing the median with interquartile range (^*^p < 0.05, ^**^p < 0.01, ^***^p < 0.001).

Our data above showed a positive anti-candidal effect with respect to surface adsorption, but also showed that a brief CUR pre-exposure (3 min) resulted in reduces adhesion of *C. albicans*, suggesting sub-inhibitory concentrations elicited some biological activity. We therefore sought to further investigate this effect on *C. albicans* by extending the CUR pre-exposure time. We were able to show that extending the time from 3 to 30 and 90 min significantly enhanced anti-adhesion (*p* > 0.01). Though, between 30 and 90 min there were no significant improvements in anti-adhesion properties (Figure 2A).

In order to understand the longer term effects of CUR treatment on *C. albicans* and how this could impact biofilm formation, we prepared yeast cells (Y = 0 min), germlings (G = 120 min) and hyphae (H = 240 min) prior to CUR exposure at 50, 100, and 200 μg/ml, which were then allowed to develop for biofilm for 24 h (Figure 2B). The resultant data showed that at lower sub-inhibitory concentrations (50 μg/ml) and PMIC levels (100 μg/ml) the anti-biofilm effects moderately impacted the overall biofilm metabolism, though at SMIC levels (200 μg/ml) a significant reduction in biofilm formation was observed for Y and G to approximately >90% of the control (*p* < 0.01), though H cells were least impacted (*p* < 0.05).

Next, we sought to determine whether CUR exhibited any additional effects on *C. albicans*. The premise of the experiment was to assess whether CUR induced aggregation through alteration of the cell wall surface. We reasoned that using a plate counting based approach of cells treated at sub-inhibitory concentrations, then if aggregation occurred then the CFU would be lower than the respective control as each aggregate would result in only one CFU due to a heterogeneous population of cells. Indeed, we demonstrated a significant reduction in cell counts in the CUR group (*p* < 0.01), which was further confirmed through light microscopy observations (Figure 2C).

### Curcumin affects the temporal expression of *Candida albicans* adhesins

**Figure 3:**
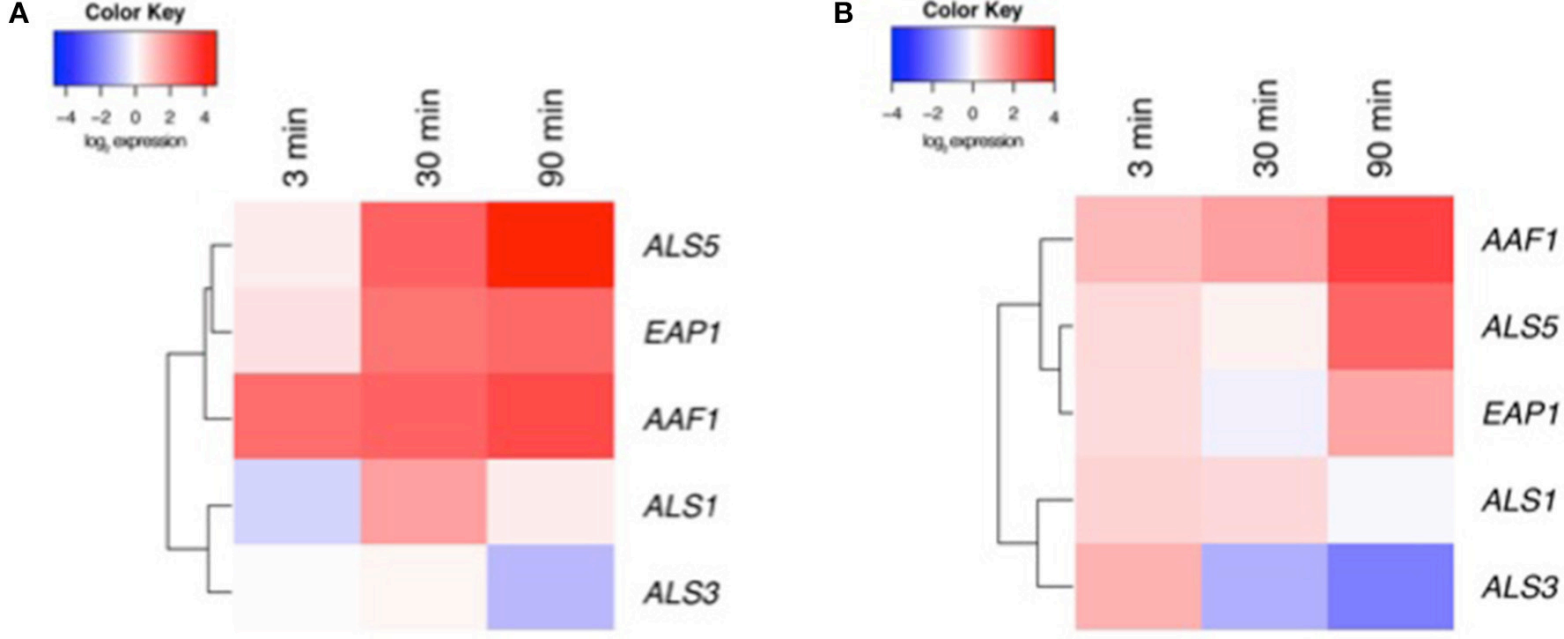
**Transcriptional analysis of CUR treated *C. albicans*. (A)** Y and **(B)** H cells were prepared and exposed ± CUR for 3, 30, and 90 min. Expression of ALS1, ALS3, ALS5, EAP1, and AAF1 were then assessed using qPCR and relative gene expression assessed the ACT1 housekeeping gene. A heatmap and clustering was created for the differential expression of genes (log_2_).

Our data above suggest that CUR elicits biological effects on *C. albicans*, most significantly on preventing biofilm formation and impacting aggregation. We therefore aimed to assess a panel of associated genes through transcriptional analysis. To do this we focussed on Y and H cells at 3, 30, and 90 min post CUR exposure. Visual representation of these patterns was illustrated using heat map analysis and hierarchical clustering (Figure 3; raw C_*T*_ data profiles are presented in Supplementary Figure 1). We demonstrated that Y cells treated with CUR showed temporal changes in gene expression, most notably the down-regulation of the adhesin ALS3, and minimal impact on its related ALS1. Whereas, the clustered aggregative and flocculation genes AAF1, EAP1, and ALS5 transcripts were all up-regulated in a time dependant manner. Similar patterns and clustering of expression were also observed for the H cells, with AAF1 showing the highest levels of expression at 30 min compared to the control, and reciprocally ALS3 being the most down-regulated. Overall though the levels of differential expression were consistently lower in the H cells (Figure 3B) than Y cells (Figure 3A).

## Discussion

CUR is a polyphenol with potent biological effects, and has been described as a modern biological regulator (Esatbeyoglu et al., 2012). The data presented herein demonstrate the potential for its use within the context of oral health for denture wearers. We have shown that CUR has the capacity to adsorb to denture relevant substrates and to inhibit *C. albicans* adhesion, rather than actively kill or inhibit the microorganisms. Interestingly, *C. albicans* exposed to CUR induced cellular aggregation, and effect that also reduced its adhesion capacity. Transcriptional analyses revealed that key adhesins were negatively impacted whereas genes associated with aggregation were positively impacted. Collectively, these data demonstrate that CUR has the potential to be used in denture care as a means of preventing denture-induced stomatitis, a disease associated with *C. albicans* biofilms (O'Donnell et al., 2015a).

Initially, we wanted to evaluate and confirm the antimicrobial properties of CUR. Our data is in general agreement with others, showing that concentrations around 100 μg/ml are required to inhibit cellular growth (Martins et al., 2009; Sharma et al., 2010; Khan et al., 2012), and 200 μg/ml to elicit any anti-biofilm activity (Shahzad et al., 2014). Any deviations can be accounted for in terms of variability of CUR source and purity, ratios of curcuminiods involved, and protocols used for broth microdilution method and the strains used. Given that our primary interest was in preventing *C. albicans* opposed to actively inhibiting and killing *C. albicans* then we focussed on lower sub-inhibitory concentrations of 50 μg/ml. This was driven through the translational possibility that CUR could be taken as part of a diet or supplement, so could be maintained within saliva as well as adsorbing to hard tissues and prostheses in the oral cavity. Here, we showed that ~6% of the CUR provided adsorped to the surface within 10 min. Conceptually, CUR could be delivered directly during ingestion, and then back through the blood into saliva indirectly. In Nepal and India for example, daily CUR consumption can reach up to 100 mg in Nepal and India (Shahzad et al., 2015), whereas in South Korea this may only reach 15 mg (Kwon, 2014). So delivering an antimicrobial concentration solely through a diet is not without challenges, though reaching an anti-adhesive concentration is possible. To this end we were able to demonstrate that 50 μg/ml could be readily adsorbed onto PMMA, the polymer used to construct denture prostheses. The optimized PMMA adsorbed with CUR was shown to reduce *C. albicans* adhesion by up to 70%, which was further reduced by 93% when a 3 min pre-treated *C. albicans* were inoculated on the optimized CUR adsorbed PMMA denture material. This synergized effect demonstrated that CUR has dual functionality, through surface adsorption and directly against *C. albicans*.

To further understand the biological basis of the sub-inhibitory CUR effect, we undertook a series of experiments to determine its effects on the kinetics of adhesion, whilst also evaluating how its impact on different morphological forms of *C. albicans*. We have previously shown that other natural compounds, such as tea tree oil (TTO) derivatives and carbohydrate-derived fulvic acid (CHD-FA) affect *C. albicans* development depending on the stage of biofilm growth (Ramage et al., 2012; Sherry et al., 2012), therefore we reasoned that CUR could also interact with *C. albicans* in a similar manner. We were able to show that prolonged exposure (30 min) of *C. albicans* yeast cells significantly reduced its adhesive capacity onto PMMA, suggesting that CUR was able to modify its adhesive capacity in some way. We further investigated this through looking at cells grown to different stages of morphological maturation, namely Y, G, and H cells. We hypothesized that depending on the stage of growth that the cells were exposed to CUR then this may affect overall biofilm development. Indeed, we showed that all morphological forms displayed reduced overall biofilm formation, though only the 200 μg/ml significantly reduced the Y and G cells in comparison to the other concentrations. H cells were not affected in a concentration dependant manner, suggesting that CUR was more effective against immature morphological forms. Interestingly, although biofilm formation was generally inhibited compared to control levels, there was still significant biofilm remaining, again suggesting early preventative intervention was most beneficial. The major limitation of this interpretation is the sample sizes used during these analyses (Vaux, 2012). Indeed, it begs the question whether the statistical analyses are worthwhile, hence why individual data-points are presented. Nevertheless, when we look at the data in its entirety, there are certainly trends suggestive that CUR exhibits positive biological effects, though further studies are required to confirm our observations.

CUR is a polyphenol with both antioxidant and hydrophobic properties (Priyadarsini, 2013; Mirzaei et al., 2017), which may explain why it preferentially adsorbs to PMMA and the cell wall of *C. albicans*. We hypothesized that the hydrophobic nature of the molecule could drive the coated *C. albicans* cells to aggregate with one another, and if we consider this within the context oral delivery within saliva then there is the possibility of creating complexes of cells that minimize their interaction with the denture surface. Indeed, we were able to demonstrate this both quantitatively and visually, which may explain why we observe synergized inhibition of adhesion at sub-inhibitory concentrations. *C. albicans* possesses a range of morphological and genetic attributes suited to colonization and biofilm formation (Blankenship and Mitchell, 2006). Finding ways of impacting these offers possibilities for novel anti-candidal therapeutics. Mechanistically, we were intrigued to understand how CUR induced *C. albicans* specific effects. Previous studies have shown that CUR has the ability to modulate of global repressor of filamentation TUP1 (Sharma et al., 2010). Indeed, our own focussed studies on mature biofilms showed that HWP1, a key hyphal wall associated element, was down-regulated (Shahzad et al., 2014). To this end we employed a transcriptional approach to assess genes implicated in adhesion and aggregation. CUR appeared to down-regulate the ALS agglutanins (ALS1 and ALS3), in both Y and H cells, suggesting it minimized their adhesive capacity. Both genes have been shown to be important in early biofilm events (Nailis et al., 2009; Fox et al., 2015). Of these ALS3 appeared to the most affected in these cells, which encodes the protein with a superior adhesive role and pivotal role in biofilm formation (Zhao et al., 2004; Nobile and Mitchell, 2005; Hoyer et al., 2008). ALS5 is a less well-defined member of this family, and although defined as an adhesin, functionally it appears to have amyloid properties and the capacity to improve aggregation (Rauceo et al., 2004; Garcia et al., 2011). This may explain why it is up-regulated following CUR treatment and this fits with the phenotype we observe. Moreover, AAF1 was also up-regulated by CUR in both Y and H cells, which is a gene highly related to the aggregation and flocculation (Fu et al., 1998). Interestingly it appears to have a minimal role in adhesion (Rieg et al., 1999), so further supports the notion of the phenotypes and anti-adhesive properties observed following induction by CUR. EAP1 showed similar trends to ALS5, though this encodes a protein known to enhance adhesion and biofilm formation (Li et al., 2007; Fox et al., 2015). This data is somewhat surprising, as we would have expected a similar level of down-regulation to that of ALS3. This suggests that EAP1, while exhibiting these adhesive properties, may have supplementary roles in cell-cell adhesion, though this requires further investigation. Collectively, these data demonstrate that CUR has the capacity at low concentrations to induce meaningful biological effects beneficial to minimizing candidal colonization.

In summary, given the strict denture hygiene regimen of brushing and cleansing required by an aging denture wearing global population, then this research provides opportunities to augment existing oral health strategies through dietary intake of important polyphenols like CUR. Not only is CUR effective against *C. albicans*, but also other oral pathogens (Shahzad et al., 2015). Given that the microbiome and mycobiome of denture wearers is highly diverse (O'Donnell et al., 2015b), then the broad-spectrum profile increases the overall appeal of this oral healthcare strategy. Indeed, there is merit to consider using a CUR based solution as a denture soak that has the potential to act and minimize further adhesion of these pathogenic biofilm related microorganisms, which with the enhanced antimicrobial activity induced through photactivation (Cieplik et al., 2015), may provide both a dual decontamination and preventative strategy. Indeed, clinical studies have already shown an additional benefit to photodynamic therapy (PDT) alone (Pereira et al., 2015), as well as alongside CUR in the context of oral health (Leite et al., 2014). Mechanistically PTD works through locally acting light-activated photoantimicrobials molecules that produce highly reactive oxygen species, which are harmful to the site of action (Wainwright et al., 2017). This approach could therefore provide an augmentative benefit in enhancing lower concentrations of orally delivered antimicrobials, which could be light-activated bi-daily or more frequently. The hurdles will be in establishing and maintaining activatable concentrations that continue to exert an anti-adhesive effect. Careful consideration on the delivery of these molecules is required, and partnering up with nanotechnological approaches seems an obvious avenue of investigation, such as the creation of nanosized curcumin, which has already been shown to improve cellular interaction (Gopal et al., 2016), and would optimize our ability to deliver biologically relevant concentrations.

## Author contributions

HA, RR, and MS participated in study design and experimental procedures and were responsible for preparation of the manuscript. DL consulted and performed the statistical analysis CW, DR and CN contributed to study design and manuscript preparation. GR and EC conceived the study, participated in study design and was responsible for producing the final manuscript. All authors have read and approved the final manuscript.

### Conflict of interest statement

The authors declare that the research was conducted in the absence of any commercial or financial relationships that could be construed as a potential conflict of interest.
