# Supplementary figures and images for: The Anti-Adhesive Effect of Curcumin on Candida albicans Biofilms on Denture Materials
Source: Front Microbiol. 2017 Apr 20;8:659. doi: 10.3389/fmicb.2017.00659 (PMC5397414; doi:10.3389/fmicb.2017.00659)

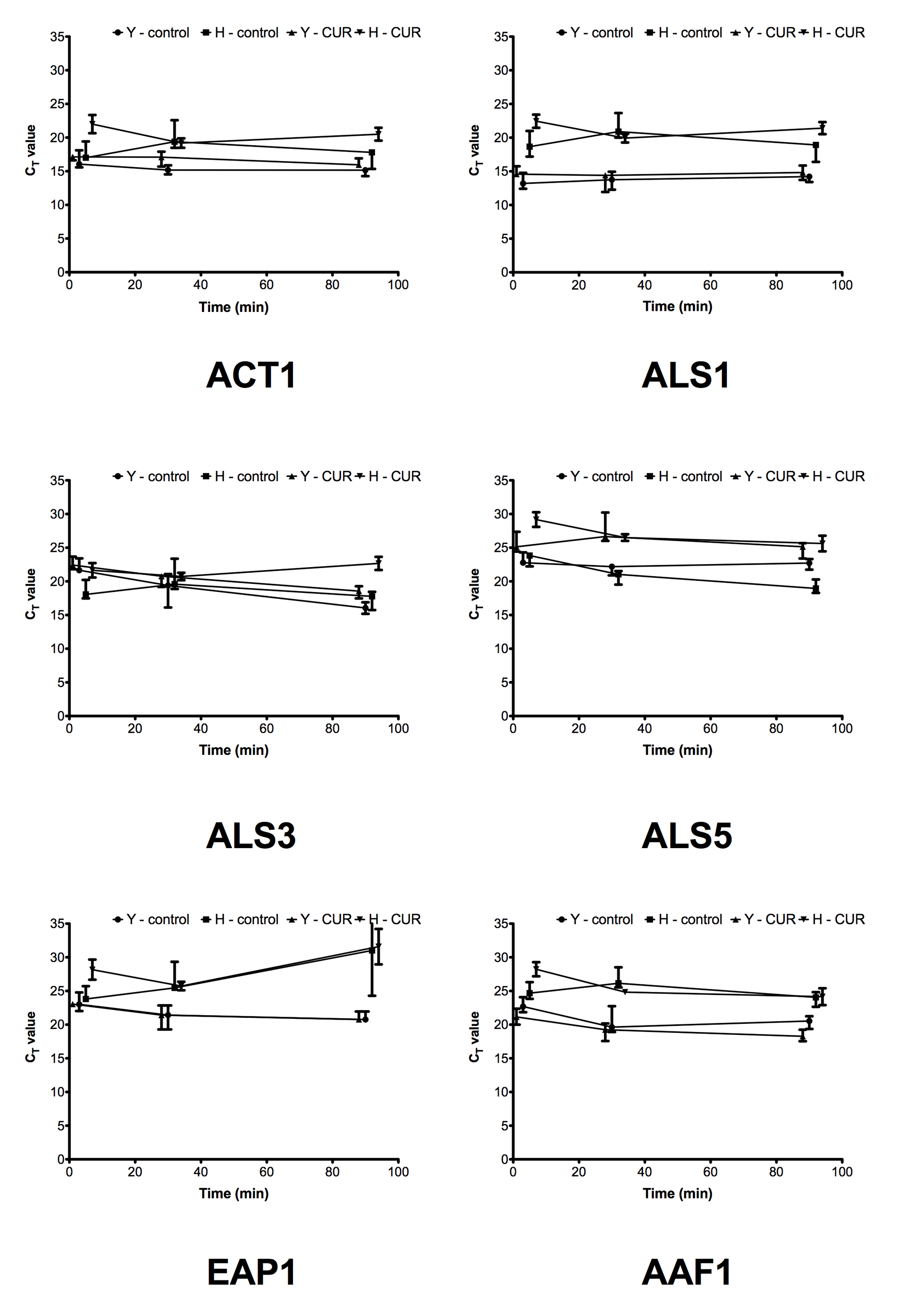

Supplement: Supplementary file 1 [file Image1.TIFF]
